# Bioaugmented Phytoremediation of Metal-Contaminated Soils and Sediments by Hemp and Giant Reed

**DOI:** 10.3389/fmicb.2021.645893

**Published:** 2021-04-20

**Authors:** Andrea Ferrarini, Alessandra Fracasso, Giulia Spini, Flavio Fornasier, Eren Taskin, Maria Chiara Fontanella, Gian Maria Beone, Stefano Amaducci, Edoardo Puglisi

**Affiliations:** ^1^Department of Sustainable Crop Production, Università Cattolica del Sacro Cuore, Piacenza, Italy; ^2^Department for Sustainable Food Process, Università Cattolica del Sacro Cuore, Piacenza, Italy; ^3^CREA – Centro Viticoltura ed Enologia, Gorizia, Italy; ^4^SOLIOMICS srl, Udine, Italy

**Keywords:** phytoremediation, bioaugmentation, heavy metals, plant-root-microbes interactions, plant uptake and accumulation, *Arundo donax* (L.), *Cannabis sativa* L., plant growth-promoting rhizobacteria

## Abstract

We assessed the effects of EDTA and selected plant growth-promoting rhizobacteria (PGPR) on the phytoremediation of soils and sediments historically contaminated by Cr, Ni, and Cu. A total of 42 bacterial strains resistant to these heavy metals (HMs) were isolated and screened for PGP traits and metal bioaccumulation, and two *Enterobacter* spp. strains were finally selected. Phytoremediation pot experiments of 2 months duration were carried out with hemp (*Cannabis sativa* L.) and giant reed (*Arundo donax* L.) grown on soils and sediments respectively, comparing in both cases the effects of bioaugmentation with a single PGPR and EDTA addition on plant and root growth, plant HM uptake, HM leaching, as well as the changes that occurred in soil microbial communities (structure, biomass, and activity). Good removal percentages on a dry mass basis of Cr (0.4%), Ni (0.6%), and Cu (0.9%) were observed in giant reed while negligible values (<100‰) in hemp. In giant reed, HMs accumulated differentially in plant (rhizomes > > roots > leaves > stems) with largest quantities in rhizomes (Cr 0.6, Ni 3.7, and Cu 2.2 g plant^–1^). EDTA increased Ni and Cu translocation to aerial parts in both crops, despite that in sediments high HM concentrations in leachates were measured. PGPR did not impact fine root diameter distribution of both crops compared with control while EDTA negatively affected root diameter class length (DCL) distribution. Under HM contamination, giant reed roots become shorter (from 5.2 to 2.3 mm cm^–3^) while hemp roots become shorter and thickened from 0.13 to 0.26 mm. A consistent indirect effect of HM levels on the soil microbiome (diversity and activity) mediated by plant response (root DCL distribution) was observed. Multivariate analysis of bacterial diversity and activity revealed not only significant effects of plant and soil type (rhizosphere vs. bulk) but also a clear and similar differentiation of communities between control, EDTA, and PGPR treatments. We propose root DCL distribution as a key plant trait to understand detrimental effect of HMs on microbial communities. Positive evidence of the soil-microbe-plant interactions occurring when bioaugmentation with PGPR is associated with deep-rooting perennial crops makes this combination preferable over the one with chelating agents. Such knowledge might help to yield better bioaugmented bioremediation results in contaminated sites.

## Introduction

Soil represents a crucial but limited resource for human activities; erosion, loss of organic matter, landslides, and contamination are critical problems that limit its utilization. Among the inorganic compounds, heavy metals (HM) have a great importance in industrial application (Lebeau et al., 2008; Rajkumar et al., 2012; Ali et al., 2013), but their release into the environment poses a serious risk to human health and other living organisms (Duruibe et al., 2007; Liu et al., 2013).

Nickel (Ni) is a heavy metal widely distributed in the environment and is released from both natural sources and anthropogenic activity (Sarwar et al., 2017). Chromium (Cr), being very resistant to corrosion is broadly utilized in various industrial applications (Emsley, 2011). Chromium is essential for living organisms, but it is toxic in excessive concentrations; in particular for humans, Cr deficiency could negatively affect lipid and sugar metabolisms (Anderson, 1997). Copper (Cu) is an essential trace element in plants and animals, but high quantities of copper salts produce acute toxicity in humans and animals (Wuana and Okieimen, 2011), possibly due to the generation of reactive oxygen species (ROS) (Liu J. et al., 2018). Cu contamination in soils could derive from natural sources like rock phosphate, from Cu-based fungicides (Komárek et al., 2010) or from zinc fertilizer application in agricultural land (Ali et al., 2013; Sarwar et al., 2017).

Traditional soil remediation technologies, such as contaminant immobilization, pollutant stabilization, soil washing, and vitrification are expensive and detrimental for the chemical properties of soil and for its biodiversity (Cunningham and Ow, 1996; Ali et al., 2013; Mahar et al., 2016). On the contrary, phytoremediation, the use of plant to immobilize or remove the contaminants in soils, is a green technology that improves chemical, physical, and organic soil properties, and that is cheaper than traditional remediation techniques (Cunningham et al., 1995; Salt et al., 1995; Barbosa et al., 2015). Several methods to improve phytoremediation efficiency have been proposed, one is the assisted phytoremediation (Lebeau et al., 2008; Shahid et al., 2014), where the availability of the contaminants is increased by the addiction of different chelating agents (CA). One of the most utilized CA to improve phytoextraction efficiency and to reduce the duration of the phytoremediation process is the ethylene diamine tetraacetic acid (EDTA), which increases the metal solubilization in soils (Shahid et al., 2014). A negative aspect of EDTA-assisted phytoremediation is the low degradability of EDTA (Lombi et al., 2001) that could be toxic for plants and animals (Lasat, 2002; Römkens et al., 2002; Evangelou et al., 2007). A promising alternative to EDTA-assisted phytoremediation can be obtained by stimulating the degradative microbial population naturally evolved in polluted soils, a process called rhizoremediation (Kuiper et al., 2004; Vergani et al., 2017; Terzaghi et al., 2019). This process can be further improved by selecting and adding to the soil selected microorganism with high degradation or biosorption abilities, an approach that can be defined as bioaugmented rhizoremediation (Lebeau et al., 2008; Rajkumar et al., 2012; Truu et al., 2015; Sarathambal et al., 2017). This technique has been proposed in case of organic pollutants (Passatore et al., 2014; Truu et al., 2015; Schwitzguébel, 2017) but only recently for heavy metals (Abhilash et al., 2012; Truu et al., 2015; Pandey et al., 2016; Tripathi et al., 2016).

Bioremediation that utilizes living organisms and/or their products to improve removal of pollutants from the environment is an emerging low-input biotechnology for ecosystem revitalization (Abhilash et al., 2012). Different microbes with plant growth-promoting traits (Tak et al., 2013), the so-called PGPR rhizobacteria (Gullap et al., 2014), have been studied for their potential to stimulate plant nutrient uptake, alleviate metal toxicity, immobilize/mobilize heavy metals in the soil, improve plant health and regulate plant pathogens (Manoj et al., 2020; Sahib et al., 2020; Prakash, 2021).

A crucial aspect in phytoremediation trials is the choice of the most appropriate plant species, as the tolerance to contaminants and accumulation capacity vary greatly among species and at times also within the same species (Pietrini et al., 2010; Shi et al., 2012). The success of phytoremediation depends on the combination of yield vs. HM uptake, for which the following crop categories have been proposed: field crops (Vamerali et al., 2010), aromatic plants (Pandey et al., 2019), *Brassica* species (Marchiol et al., 2004; Mourato et al., 2015), hyperaccumulator plants (Cheng, 2003; Peer et al., 2006) and biomass crops, either annual or perennial (Shi and Cai, 2009; Pandey et al., 2016; Tripathi et al., 2016).

Here in thus study, we choose two model non-food high-yielding crop for phytoremediation to address the contamination by Cr, Zn, and Cu of two distinct environmental matrices: hemp for soil and giant reed for sediment. Hemp (*Cannabis sativa L.*) can tolerate high heavy metal content in soil (Angelova et al., 2004), and it can be considered a good candidate crop in phytoremediation experiments (Linger et al., 2002; Rheay et al., 2020) because of its fast growth (Struik et al., 2000), HM stress tolerance genes (Ahmad et al., 2016), and fine and deep rooting systems (Amaducci et al., 2008). Phytoremediation with hemp permits to produce biomass for multipurpose bioenergy applications (Amaducci et al., 2015; Rheay et al., 2020), simultaneously with the reduction of soil contaminants (Citterio et al., 2003; Linger et al., 2005). Giant reed (*Arundo donax* L.) is a perennial plant with a high biomass yield in marginal land with low inputs (Amaducci et al., 2017), high belowground C storage potential (Martani et al., 2020) and is tolerant to heavy metals (Papazoglou et al., 2005, 2007; Yang et al., 2012; Barbosa et al., 2015; Cristaldi et al., 2020) thanks to its ability to store HMs in belowground organs (Fiorentino et al., 2017). Giant reed especially in wetland and sediments along riverbanks, where it naturally grows (Barney and DiTomaso, 2008; Nackley et al., 2013), is a good candidate for phytoremediation (Mirza et al., 2010; Bonanno, 2012; Truu et al., 2015).

Contaminated soil and sediments from an industrial area of Northern Italy were used to isolate, screen, and select metal-chelating plant growth-promoting bacteria. A pot experiment on the same soil was performed to compare the phytoremediation potential of both traditional (crop alone) and assisted phytoremediation techniques (PGPR and chelating agent) in order to quantify (1) HM uptake and (2) understand the plant-soil-microbe interactions. We hypothesized that bioaugmentation with PGPR, more than with chelating agents, can alleviate HM stress on plant growth of these two high-yielding non-food crops and its combination with such deep-rooted crops can help in increasing their phytoremediation potential of HM-contaminated soils and sediments. If decreasing stress on root growth and plant photosynthesis this should led to (1) a fine root system more similar to those of the same crops grown on non-contaminated matrices, (2) a higher HM uptake in plant tissues, and (2) a less marked effect of HMs on the microbial community structure and activity of rhizosphere soil.

## Materials and Methods

### Soil Collection Site and Plants Preparation

Surface soil (0–60 cm) and sediment (0–30 cm) for pot experiment were collected within the polluted area around an industrial site operating in the sector of plastic galvanization in Northern Italy. Contaminated and non-contaminated soil and sediments were collected respectively 100 m after and before a factory’s discharge point into the river. Both soil sites (dystric cambisols) are hay meadows while sediment has been colleted within the 5-m-wide sandy flood bed of the river. Soil and sediment samples were air dried and then sieved at 8 mm, mixed, and homogenized, then aliquots were further sieved at 2 mm to eliminate the skeleton and analyzed for their main physiochemical properties and HM levels (Table 1). Soil samples were mainly contaminated by Cr, Ni, and Cu while sediment mainly by Cu according to the Italian legislative limits for public areas (Table 1). Giant reed (*Arundo donax L*.) rhizomes to be grown in sediments were collected from a 9-year field trial (Ferrarini et al., 2020), washed, cut into 3-cm-length pieces and precultivated in peat for 7 days using a modified Hoagland solution to check for growth rate homogeneity. Hemp (*Cannabis sativa* L.) seeds of commercially available variety Futura (Hemp-it, France) were used in the experiment.

**TABLE 1:** Main physiochemical parameters of for contaminated (C) and non-contaminated (NC) soil and sediments and their total chromium (Cr), nickel (Ni), and copper (Cu) concentrations (mg kg^–1^) at the beginning of the experiment.

	Sand	Silt	Clay	Texture class	Field capacity	Wilting point	SOM	N tot	C/N	pH	CaCO_3_	Cr*	Ni*	Cu*
							
	%	%	%		%	%	%	%			%	mg kg^–1^		
NC soil	47	31	23	Loam	26	14	1.9	0.09	12.1	6.9	1.5	19.1	21.7	41.7
Contaminated soil	45	38	17	Loam	26	13	2.0	0.10	11.3	6.8	2.0	97	**526**	**172**
NC sediment	87	9	4	Sand	13	5	0.9	0.07	7.5	7.4	3.0	24.8	26.8	39
Contaminated sediment	83	12	5	Loamy sand	14	5	1.1	0.08	7.9	7.3	3.2	33.5	**133.2**	64.9

Bold values above the screening values are values established for residential soil use by the Italian Ministry of Environment (DM 152/2006). *Legislative limits currently adopted in Italy (DM 152/2006) for HM concentration in soil for green areas/residential use are respectively 150, 120, and 120 mg kg^–1^ for Cr, Ni, and Cu.

### Isolation, Screening, and Selection of Metal-Chelating Plant Growth-Promoting Bacteria

A sequential screening approach was carried out in order to select from the contaminated soil and sediment bacterial strains with the ability to grow under selective pressure of Zn, Cu, and Cr; the resulting strains were then screened and quantified for plant growth-promoting traits (P solubilization), minimum inhibitory concentrations (MICs), and biosorption abilities toward the three tested metals.

Isolations were carried out using three replicates of the same contaminated soil and sediment used for greenhouse pot experiment. Ten grams of soil or sediment were added with 100 ml of sterile physiological solution and placed on a horizontal shaker for 24 h. The obtained slurries were then diluted decimally and plated on tryptone soy agar (TSA) plates containing 100 ppm of each metal as NiCl_2_⋅6H_2_O, CuSO_4_, and CrCl_3_⋅6H_2_O salts (Carlo Erba reagents, RPE, analytical reagent grade). Representative colonies were picked and dereplicated with random amplified polymorphic DNA (RAPD) amplification as detailed in Spini et al. (2018).

The resulting unique strains were then screened for their phosphate solubilization abilities by spotting them on GY/tricalcium phosphate medium containing Ca_3_(PO_4_)_2_ as insoluble source of phosphorus: the plates were incubated at 30°C, and after 7 days, the P solubilization ability was quantified by measuring the halos diameter as previously described (Ambrosini and Passaglia, 2017; Guerrieri et al., 2020). Isolates without a halo were considered non-solubilizers (−); isolates with a halo between 1 and 2 cm as level 1 (++); isolates between 2 and 3 cm as level 2 (++); and isolates with > 3 cm as level 3 (+++).

The ability of all isolates to withstand increasing metal concentrations was quantified with a modification of the MIC method usually applied for antibiotic. Each strain was grown overnight in tryptone soy broth (TSB), and 100 μl of a 1/10 dilutions were dispensed in 96-well microplates together with 100 μl of TSB at increasing concentrations of 0, 200, 400, 800, 1,600, and 3,200 ppm of NiCl_2_, CuSO_4_, and CrCl_3_. Each strain was tested in 10 replicates.

A screening was carried out by selecting strains that had a P solubilizing ability of level 1 or higher and the ability to withstand the metal mixture with a MIC higher than 200 ppm concentration. The retained strain were then tested for their metal biosorption abilities with the method described by Ma et al. (2015). Briefly, strains grown overnight in TSB were washed twice with distilled water and resuspended in 2 ml of distilled water containing 200 ppm of NiCl_2_, CuSO_4_, and CrCl_3_. After 8 h of incubation at 30°C, the tubes were centrifuged and the unsorbed metals remaining in the cells free supernatant quantified as described below. The bacterial pellet was dried and weighted to measure the biomass and normalize accordingly the data obtained.

Two strains showing the best biosorption abilities were finally selected from the soil and the sediment batches respectively, and were identified by means of Sanger sequencing of 16S rRNA PCR amplicons as described in Spini et al. (2018). The amplification was carried out using the primers P0 (5′-GAG AGT TTG ATC CTG GCT-3′) and P6 (5′-CTA CGG CTA CCT TGT TAC-3′) described in Di Cello and Fani (1996) prior to their use in the microbial-assisted phytoremediation experiments; the two strains were grown overnight in TSB to an exponential phase. They were then washed twice with distilled water and finally resuspended in distilled water to an OD at 600 nm as determined by UV-VIS Spectrophotometer (AT1409001, Aurogene, Italy) corresponding to 10^10^ CFUs L^–1^. In the PGPR-treated thesis, the resuspension was added to the soil pots in order to reach a final bacterial load of 10^8^ CFUs kg^–1^ of soil.

### Greenhouse Pot Experiment

The comparison of the phytoremediation potential with common practices (crop alone) as compared with assisted phytoremediation techniques (PGPR and chelating agent) was conducted through a pot experiment performed under controlled conditions. Hemp seeds and rhizome of giant reed were transplanted into filled pot of 16 L (60 cm height, 16 cm diameter) and cultivated 60 days (25:16°C day:night temperature, with a photoperiod of 16 h). One rhizome/seed were transplanted/sown per pot. Pot dry weights were respectively 6 and 7.2 kg for giant reed and hemp, respectively. Soil has been maintained at 60% of water holding capacity while sediment at 100% WHC to simulate sediment water conditions. This implies that the findings derived from this study will require field validation. In particular, sediment pots with a collecting tube for leaching were built at the bottom of the top to collect leachate solutions before and after treatments. The use of, e.g., EDTA mobilizing agents, indeed, in field trials is sensitive and requires adequate greenhouse evaluation prior to upscaling to the field scale. Four main treatments (*n* = 4 replicates) were applied to sediment and soil respectively grown with giant reed and hemp (*n* = 32 pots, Supplementary Figure 1): not contaminated with crop alone—control (NC), contaminated with crop alone (C), contaminated and treated with PGPR (C+PGRP), and contaminated and treated with EDTA (C+EDTA). Three additional pots for both soil and sediment (C and NC) were kept for the duration of the experiment without plant to characterize microbial diversity without plants (Supplementary Figure 1). Pots were inoculated with PGPR and irrigated with EDTA twice during the experiment: 22 and 51 days after transplanting (DAT) for the giant reed pots and 34 and 53 days after sowing (DAS) for the hemp pots. PGPR were inoculated *via* irrigation of pots of 1 L solution of 10^8^ UFC ml^–1^ of selected bacteria strain (Section “Isolation, Screening, and Selection of Metal-Chelating Plant Growth-Promoting Bacteria”). EDTA (Carlo Erba reagents, RPE, analytical reagent grade) were applied as 1 L solutions at 0.5 g kg^–1^ concentration as suggested by Shahid et al. (2014) for the same HMs and C3 plants. Both solutions were inoculated at the end of the lighting period in order to allow the plants to adapt to the solutions and to show the inoculation effects on photosynthetic performances the following day.

### Plant and Soil Sampling for Pot Experiment Monitoring

#### Heavy Metals Determination on Plant, Soil, and Leachate Samples

Leachate solution were collected in 1 L flask from the bottom of the *n* = 16 giant reed pots 17, 26, 38, and 60 DAT solutions were immediately filtered at 0.45 μm and stored at −18°C until analysis.

At the end of the experiment (78 DAT and respectively 56 and 28 days after first and second applications), aboveground (ABG) and belowground (BGB) biomass were harvested from all pots (*n* = 32). Leaves and stems were sampled separately for giant reed, while hemp samples were sampled from leaves, stem, and flowers. Aboveground biomass sample were dried at 65°C to determine dry matter content and then samples were milled and sieved at 1 mm for HM analysis. Belowground samples (roots for hemp and rhizome+root for giant reed) were carefully washed with distilled water before root analysis (Section “Fine Root System Characterization”).

The rhizosphere soil (RS) was collected for each pot according to previously described methodology (Barillot et al., 2013; Marasco et al., 2018; Guerrieri et al., 2020). Briefly, bulk soil (BS) was removed by shaking plants by hand for 10 min vigorously, paying attention to the roots’ integrity, as long as the roots’ non-adhering soil particles were completely removed. In order to collect rhizosphere soil, the root system was washed with 500 ml of 0.9% NaCl added and afterward 150 ml of bacterial suspension were incubated at 25°C for 90 min with shaking at 180 rpm. BS and RS were immediately dried at 65°C for HM analyses and stored at −18°C until soil enzyme activities and DNA extraction for bacterial diversity analysis. Soil and sediment total Cr, Ni, and Cu concentrations were analyzed at beginning (*T*_*z*__*ero*_) on BS samples and at the end of experiment (*T*_*f*__*inal*_) on either BS and RS samples. Soil and sediment samples were digested with a solution of aqua regia (HCl:HNO_3_ in a volume ratio 3:1) and heated under reflux, after pretreatment with H_2_O_2_; Ni, Cr, and Cu concentrations were determined in all samples by graphite furnace atomic absorption spectrometry (GFAAS) (Perkin-Elmer AA-600).

Above and belowground dry samples (1 g subsamples from each individual pot sample) were analyzed for total Ni, Cr, and Cu concentrations as in Watanabe et al. (2015). The samples were digested in a solution of 6 ml of concentrated HNO_3_ and 1 ml of H_2_O_2_, the solution was heated at 110°C for 2 h, and then distilled water was added to reach the volume of 50 ml, the solution was filtered at 0.45 μm and then read with ICP-MS (Agilent 7900).

To assess the performance of the phytoextraction-assisted bioaugmentation with PGPR and addition of chelating agents, the following factors (Lebeau et al., 2008; Ali et al., 2013) were calculated for hemp and giant reed: plant biomass (mg pot^–1^), concentration (mg kg^–1^) and amount of metal extracted by plants (μg pot^–1^ in each plant component), bioconcentration (BCF), and translocation (TF) factors defined, respectively, as the metal in AGB to the metal in soil ratio and the metal in AGB to the metal in BGB ratio. BGB in hemp was only roots while root and rhizomes in giant reed.

#### Whole-Canopy Gas Exchange Measurements

The day before the first EDTA/PGPR application, hemp and giant reed pots were placed into a semi-automated gas exchange platform to measure whole-canopy gas exchanges for 7 days. Canopy net assimilation rate (Pn) and transpiration rate (E) were determined with a self-assembled multichamber gas exchange apparatus (fully described in Fracasso et al., 2017). In brief, in this system, air is drawn from outside and blown into the chambers while a CIRAS-DC double-channel absolute CO_2_/H_2_O infrared gas analyzer (PP-System) combined to a datalogger measures continuously, 24 h day^–1^, CO_2_ and H_2_O concentrations at the inlet and outlet of each chamber. Pn and E were calculated from flow rates and CO_2_ and water vapor differentials using the formula provided by Long and Hällgren (1993).

#### Fine Root System Characterization

Once cleaned, roots were hand recovered from the water using a 2-mm mesh sieve. Determination of root length density (RLD, cm cm^–3^) and root diameters was performed with the software winRHIZO Pro 2019. The images were acquired using the TWAIN interface at 600 dpi and with a scanner (model: Epson Expression 10000xl) equipped with a double light source to avoid roots overlapping. Fine roots dry biomass weight was determined gravimetrically, after taking scanned images, drying the roots at 60°C until constant weight. The dried fine root sample were then analyzed for total HM concentration as in Section “Heavy Metals Determination on Plant, Soil, and Leachate Samples.” The diameter class length (DCL, mm cm^–3^) was calculated for very fine (0.0–0.5 mm), fine (0.5–2 mm), and coarse (>2 mm) diameters for both crops. The DCL was calculated for 13-diameter classes from 0 to 3.15 mm (with a 0.15-mm increase per class). To describe crops’ DCL distribution as affected by treatments, the DCL data of hemp and giant reed were fitted with the non-linear regression extreme value model (Curve expert Professional 2.6.4) as suggested by Zobel et al. (2007) and successfully applied to biomass crops by Chimento and Amaducci (2015):

$$DCL\left(mm/cm^{3}\right)=a+be^{-e\left[\left[-\frac{x-c}{d}\right]-\frac{x-c}{d}+1\right]}$$

where *x* refers to diameter class (mm). In general, the coefficient *a* (baseline) is the value approached by DCL as *x* approaches positive or negative infinity, *b* is the DCL peak value minus *a*, *c* is diameter class at peak value (the *x*-axis location of *b*), and *d* (amplitude of the curve) is related to the width across the curve at half maximum (*b* / 2 + *a*) so that width at half maximum equals 2.446 *d* (Zobel et al., 2007).

#### Microbial Biomass and Enzyme Activities of Bulk and Rhizosphere Soils

Twenty soil enzymatic activities (EA) involved in key steps of soil C, N, P, and S cycling were measured: (i) α-glucosidase (agluc, EC 3.2.1.20), β-glucosidase (bgluc, EC.3.2.1.21), α-galactosidase (alfaGAL, EC 3.2.1.22), β-galactosidase (betaGAL, EC 3.2.1.23), α-mannosidase (alfaMAN, EC 3.2.1.24), β-mannosidase (betaMAN, EC 3.2.1.25), β-D-glucuronidase (uroni, EC 3.2.1.31); β-1,4-glucanase (cell, EC 3.2.1.4), β-1,4-xylanase (xilo, EC 3.2.1.8) involved in C cycling; (ii) *N*-acetyl-b-D-glucosaminidase (chit, EC 3.2.1.14), leucine amino-peptidase (leu, EC.3.4.11.1.), trypsin-like protease (tryp, EC 3.4.21. 4) involved in N cycling; (iii) acid (acP, EC.3.1.3.2) and alkaline phosphomonoesterase (alkP, EC.3.1.3.1), phosphodiesterase (bisP, E.C.3.1.4.1.), pyrophosphodiesterase (piroP, EC.3.6.1.9.), inositol-P phosphatase (inositP, EC 3.1.3.25) involved in P cycling; (iv) arylsulfatase (aryS, EC.3.1.6.1.) involved in S cycling; and (v) non-anoate (nona) and palmitate (palmit) esterase (EC 3.1.) involved in the hydrolysis of ester bonds. EA were determined on soil extracts (Bardelli et al., 2017) using fluorogenic substrates containing 4-methyl–umbelliferyl (MUF) and 7-amino-4-methyl coumarin (AMC) as fluorophores. Soil enzymes were desorbed by heteromolecular exchange procedure *via* bead-beating according to Ferrarini et al. (2020). Soil microbial biomass was determined as double-strand DNA (dsDNA) content (Fornasier et al., 2014).

#### Molecular Analyses of Bulk and Rhizosphere Bacterial Diversity

Soil and rhizosphere samples from the hemp and the giant reed experiments were collected at the beginning and at the end of the experiments and analyzed in quadruplicates for bacterial diversity by means of high-throughput sequencing (HTS) of 16S rRNA amplicons. The procedure applied is described in detailed in Spini et al. (2018) and summarized as follows.

Total microbial DNA was extracted from 0.5 g of each soil sample with the Fast DNA^TM^ SPIN Kit for Soil (MP Biomedicals, United States) with the number of modifications: homogenization in the FastPrep^®^ for 40 s at speed setting of 6.5 twice, keeping in ice between the two homogenization steps, final centrifugation at 14,000 × *g* for 15 min, and the final resuspension of the binding matrix was carried out in 50 μl^–1^ of nuclease-free water. The DNA quality of each sample was checked with electrophoresis on a 1% agarose gel, and QuBit^TM^ fluorometer (Invitrogen, United Kingdom) quantification was carried out in order to dilute each DNA sample to 1 ng μl^–1^ concentration. PCR amplifications of the V3-V4 regions of bacterial 16S rRNA were carried out using the universal primers 343f (5′-TACGGRAGGCAGCAG-3′) and 802r (5′-TACNVGGGTWTCTAATCC-3′) (Połka et al., 2015). Amplifications were carried out in two steps, a first with untagged primers in order to reduce the possibility of preferential primers annealing (Berry et al., 2011) and a second step using a dedicated forward primer with a 9-base extension at the 5’ end, which acts as a tag, in order to make simultaneous analyses of all samples in a single sequencing run possible. The PCR reaction mix is composed of 20.5 μl of MegaMix (Microzone Limited, United Kingdom), 1.25 μl of each primer (10 μM), and 2 μl (1 ng μl^–1^ concentration) of DNA template. Thermal cycling conditions were as follows: Step 1: an initial denaturation at 94°C for 5 min, followed by 25 cycles at 94°C for 30 s, 50°C for 30 s, 72°C for 30 s, followed by a final extension at 72°C for 10 min. Step 2: initial hold at 95°C for 5 min, followed by 10 cycles of 95°C for 30 s, 50°C for 30 s, and 30°C for 30 s; then, a final extension at 72°C for 10 min. The DNA amplifications were checked with electrophoresis on a 1% agarose gel, and then quantified using a QuBit^TM^ fluorometer (Invitrogen, United Kingdom). PCR products generated from the second step were multiplexed as a single pool using equivalent molecular weights (20 ng). The pool was then purified using the solid-phase reversible immobilization (SPRI) method with Agencourt AMPure XP kit (REF A63880, Beckman Coulter, Milan, Italy), then sequenced by Fasteris S.A. (Geneva, Switzerland). The TruSeq DNA sample preparation kit (REF 15026486, Illumina Inc., San Diego, CA, United States) was used for amplicon library preparation, whereas the sequencing was carried out with the MiSeq Illumina instrument (Illumina Inc., San Diego, CA, United States) generating 300 bp paired-end reads.

High-throughput sequencing data filtering, multiplexing, and preparation for subsequent statistical analyses were carried out as previously detailed (Vasileiadis et al., 2015). Paired reads were assembled to reconstruct the full V3-V4 amplicons using the FLASH assembler (Magoč and Salzberg, 2011), and samples were demultiplexed according to their tag using SeqKit (Shen et al., 2016). Further screenings were carried out with Mothur (Schloss et al., 2009) in order to remove sequences with large homopolymers (≥10), sequences that did not align within the targeted V3-V4 region, chimeric sequences, and sequences not classified as bacterial. Sequence data were submitted to the National Centre for Biotechnology Information Sequence Read Archive (BioProject PRJNA687540).

### Statistical Analyses

Heavy metal uptake and concentration data were analyzed separately for both crops and their relative plant components using a one-way ANOVA with treatment (C, C+PGPR, C+EDTA) as main effect. HM concentration in leachate and Simpson diversity index were analyzed using a two-way mixed-model ANOVA for complete a randomized design. Treatment combination and sampling time (*T*_*z*_, *T*_*f*_) and their interaction were considered fixed main effects with replicates as a random effect.

Pn and E data were analyzed *via* one-way ANOVA and, when the *F*-test was significant, mean separation was performed by the *t*-test at *P* < 0.05 and *P* < 0.01. Degree of variation around means is given as standard error (SE). All ANOVA were performed with *agricolae* R package while *post hoc* men separation *via multicomp* R package.

To determine whether treatments influence fine root systems, especially whether roots become thinner or thicker in response to treatments, the statistical significance of the DCL curve parameters (*a*–*d*) were assessed through testing their standard errors using the t-statistics at *P* < 0.05. Relatively to 16S, Mothur and R were employed to analyze the resulting high-quality sequences following the operational taxonomic units (OTUs) at 97% similarity, and the taxonomy-based approach, which was implemented using an amended version of the Greengenes database (McDonald et al., 2012). Sequence data were submitted to the National Centre for Biotechnology Information Sequence Read Archive (BioProject PRJNA687540). Soil EA and OTU from microbial sequencing were analyzed through multivariate analysis (distance-based redundancy analyses (dbRDA)) while OTU was also analyzed with hierarchical clustering. We used Mothur and R for statistical analyses on OTU and taxonomy matrixes using hierarchical clustering with the average linkage algorithm at different taxonomic levels. dbRDA was run on a three step basis (Ferrarini et al., 2020) separately for soil type (BS, RS) of hemp and giant reed: (1) Bray–Curtis dissimilarity (non-linear) matrix is calculated on square root transformed data for soil EA and raw data for OTU database; (2) stepwise multiple regression was performed to select the best model (AIC) including environmental variables only for soil EA data and OTU database of RS; (3) a principal coordinate analysis (PCoA) is calculated based on the distance matrix (999 permutations) to obtain dbRDA axis coordinates for main treatments (treatment for soil EA data and Trt × sampling time for bacterial diversity data) to be plotted as multivariate centroids surrounded by 95% confidence interval ellipsoids and coordinates of species (only for soil EA) and environmental variables (RLD and soil HM concentrations) respectively as points and arrows; (3) one-way permutational multivariate analysis of variance (PERMANOVA) based on Bray-Curtis matrix was conducted for 9,999 permutations was used to test for main treatment effects on soil EA and sequencing data with replicate as random effect. Planned contrasts of PERMANOVA, according to Bonferroni’s test (*P* > 0.05) were set as follows: treatment vs. soil EA grouped by element cycle (C-, N-, P-, and S-cycling, and esterases) and all contrasts for treatment × sampling time interaction terms in the case of PERMANOVA on sequencing data. A fourth dbRDA step was only run for soil EA data (Mattarozzi et al., 2020). Briefly, a similarity percentage (SIMPER) was used to select the soil EA accounting for > 90% of cumulative dissimilarity between each of all planned contrasts for main treatments (NC, C, C+EDTA, C+PGPR). dbRDA, PERMANOVA, and SIMPER analysis were run by using *vegan* R packages (*capscale*, *pairwise.adonis* and *simper* functions, respectively).

## Results

### Selection of Bacterial Strains for Microbial-Assisted Phytoremediation

Isolation and molecular fingerprint genotyping resulted in a total of 42 unique strains: 22 derived from the contaminated sediments and 20 from the contaminated soil. The assessment of phosphate solubilization ability and MIC for the three tested metals are reported in Table 2, together with measurement on biosorption abilities on 12 selected strains. Seven out of 22 strains from soil had P solubilization ability, with one (strain So17) having to generate a larger halo. Regarding sediment strains, 12 out of 17 had P solubilization abilities, with one as well having level 3. The ability to withstand high metal concentrations was confirmed by MIC values, that in most cases had values of 800 ppm or more, thus much higher than the selective concentration used in the isolations. According to the results obtained for P solubilization and MIC, 12 strains (six from soil and six from sediments) were selected for the measurement of metal biosorption ability, an important trait to improve the phytoremediation potential. Data were normalized per gram of dry cells and showed values between 0.1 and 13.5 mg of metal per gram of dry cell biomass (Table 2). The highest biosorption levels were found for strains So17 among soil isolates and strain Se02 among sediment isolates: interestingly those were also among the three strains that had the highest P solubilization abilities. Strains So17 and Se02 were thus selected for the microbial-assisted phytoremediation experiments and taxonomically identified as *Enterobacter* spp. (So17) and *Enterobacter asburiae* (Se02) (GenBank submission SUB9058427).

**TABLE 2:** Screening of isolated strains for P solubilization ability, minimum inhibitory concentrations (MICs) of Cr, Cu, and Ni and biosorption (BS) toward the three testes metals.

Strain	P solubilization^*a*^	MIC (mg L^–1^)	Cr BS (mg g^–1^_*dry cells*_)	Cu BS (mg g^–1^_*dry cells*_)	Ni BS (mg g^–1^_*dry cells*_)
So_01	+	800	1.14	0.9	2.3
So_02	-	400	nd	nd	nd
So_03	+	800	5.88	8.19	2.08
So_04	-	800	nd	nd	nd
So_05	-	400	nd	nd	nd
So_06	-	1600	nd	nd	nd
So_07	-	800	nd	nd	nd
So_08	+	800	5.59	8.73	3.55
So_09	++	200	nd	nd	nd
So_10	-	400	nd	nd	nd
So_11	-	800	nd	nd	nd
So_12	+	800	4.13	7	3.25
So_13	-	800	nd	nd	nd
So_14	-	400	nd	nd	nd
So_15	-	800	nd	nd	nd
So_16	+	400	0.133	3	0.17
**So_17**	**+++**	**800**	**3.25**	**5.42**	**1.67**
So_18	-	800	nd	nd	nd
So_19	-	400	nd	nd	nd
So_20	-	800	nd	nd	nd
So_21	+	1600	nd	nd	nd
So_22	-	800	nd	Nd	nd
Se_01	++	800	4.64	6.62	2.13
**Se_02**	**+++**	**400**	**7.5**	**13.5**	**2.1**
Se_03	+	800	4	5.5	0.9
Se_04	+	800	nd	Nd	nd
Se_05	+	800	nd	Nd	nd
Se_06	+	1600	nd	Nd	nd
Se_07	+	1600	nd	Nd	nd
Se_08	+	1600	nd	Nd	nd
Se_09	+	800	nd	Nd	nd
Se_10	++	800	4.15	5.65	0.23
Se_11	+	400	0.5	1.67	1.5
Se_12	-	400	nd	Nd	nd
Se_13	-	400	nd	Nd	nd
Se_14	-	400	nd	Nd	nd
Se_15	-	200	nd	Nd	nd
Se_16	-	200	nd	Nd	nd
Se_17	+	800	nd	Nd	nd
Se_18	++	400	1.75	2.75	0.25
Se_19	-	800	nd	Nd	nd
Se_20	+	800	nd	Nd	nd

*The two strains finally selected for the phytoremediation experiments are highlighted in bold. ^a^Data for P solubilization abilities are categorized in four groups: non-solubilizers (no halo on GYT medium); + level 1 solubilizers (halo between 1 and 2 cm) as level 1; ++ level 2 solubilizers (halo between 2 and 3 cm); +++ ++ level 2 solubilizers (halo > 3 cm).*

### Phyto-Assisted Bioremediation Performances for Cr, Ni, and Cu

#### Heavy Metals Accumulation in Plant Organs of Hemp and Giant Reed

Heavy metal concentration and total concentration of heavy metals (Cr, Ni, and Cu) in BS at the end of the experiment generally did not decrease (Supplementary Figure 2). Only Ni were significantly lower with hemp treated with EDTA and PGRP (*F* = 12, *P* = 0.04). Plant yield was significantly affected by HM pollution in sediment (−19%) and soil (−16%) as shown by tolerance index (TI) values (Table 2). Bioaugmentation with PGPR significantly alleviate HM stress on plant yield showing no difference in plant yield compared with non-contaminated sediment (NC). Giant reed and hemp treated with PGPR showed a TI of 117 and 89%, respectively.

**FIGURE 1:**
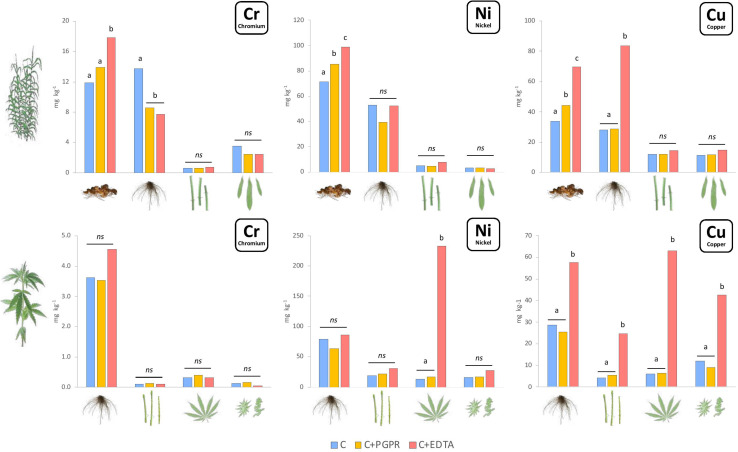
Mean values of Cr, Ni, and Cu concentration (mg kg^–1^) in plant components for hemp (roots, stems, leaves, and flowers) and giant reed (rhizome, roots, stems, and leaves) as affected by treatments. Different letters denote statistically different (Tukey’s test, *P* = 0.05) concentration values among treatment for each HM/crop combination.

**FIGURE 2:**
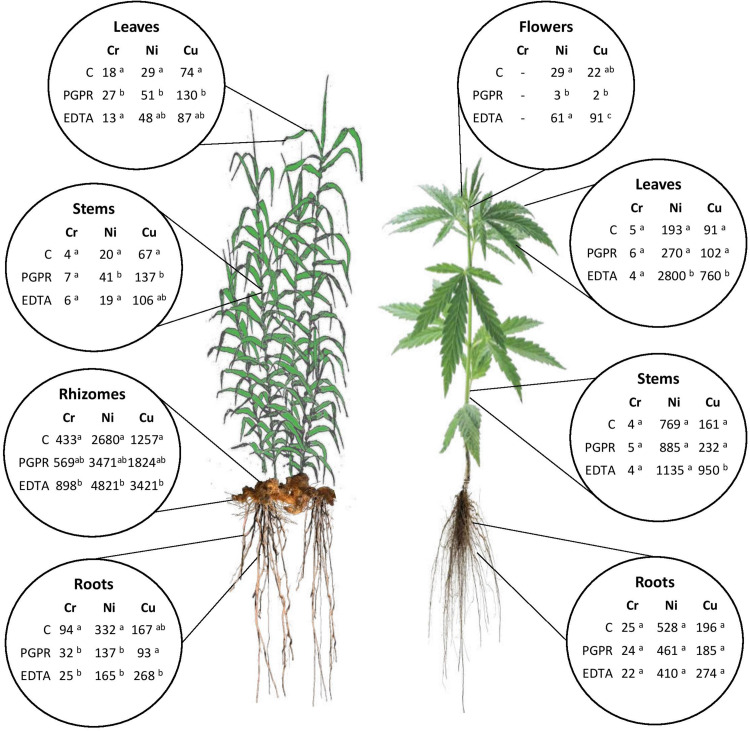
Summary of mean values of Cr, Ni, and Cu uptake (μg HM plant^–1^) in plant components for hemp and giant reed as affected by treatments. Different letters denote statistically different (Tukey’s test, *P* = 0.05) uptake values among treatment for each HM.

Heavy metals content (Figure 1) and uptake (Figure 2) were significantly enhanced by bioaugmentation with PGPR and addition of EDTA chelating agent in contaminated soil and sediments. Bioconcentration (BCF) and translocation (TF) factors of chromium, nickel, and copper in the belowground and aboveground of giant reed and hemp were depicted in Table 3. The three HMs assessed in this study showed clear distant accumulation (Figure 1) and uptake (Figure 2) patterns among plant organs.

**TABLE 3:** Bioconcentration (BCF) and translocation (TF) factors and tolerance index (TI) for hemp and giant reed.

Crop	Treatment	BCF-ABG			BCF-BGB*			TF			TI
		Cr	Ni	Cu	Cr	Ni	Cu	Cr	Ni	Cu	%
Giant Reed	C	0.084 a	0.04 a	0.32 a	0.52 a	0.59 ab	0.85 a	0.16 b	0.07 a	0.38 a	59 a^†^
	C+PGPR	0.052 b	0.03 a	0.26 a	0.38 a	0.53 a	0.82 a	0.14 b	0.07 a	0.32 a	117 b
	C+EDTA	0.051 b	0.05 a	0.36 a	0.42 a	0.68 b	1.87 b	0.13 b	0.07 a	0.19 b	66 a^†^
	**Mean**	**0.062**	**0.04**	**0.32**	**0.44**	**0.60**	**1.18**	**0.15**	**0.07**	**0.30**	**81**
Hemp	C	0.003 a	0.03 a	0.08 a	0.07 a	0.24 a	0.32 a	0.05 a	0.13 a	0.27 a	78 a^†^
	C+PGPR	0.004 a	0.04 a	0.08 a	0.07 a	0.21 a	0.30 a	0.06 a	0.19 a	0.28 a	89 b^†^
	C+EDTA	0.003 a	0.29 b	0.49 b	0.08 a	0.29 a	0.65 b	0.03 a	1.07 b	0.77 b	84 ab^†^
	**Mean**	**0.003**	**0.12**	**0.22**	**0.07**	**0.25**	**0.42**	**0.05**	**0.46**	**0.44**	**84**

Different lowercase letters indicate significant differences (Tukey’s test P < 0.05) among treatments for each factor. ANOVA has been run separately for giant reed and hemp. ABG, aboveground biomass; BGB, belowground biomass. *BGB of Giant reed is the sum roots and rhizome. ^†^ABG average value of C-treatment differed significantly from NC-treatment (non-contaminated matrix).

All plant organs showed Cr, Ni, and Cu accumulations (Figure 1). Among organs, the concentration trend was belowground organs (rhizomes, roots) > > aboveground organs (leaves, stems) for Cr and Ni whereas Cu showed similar concentration in belowground and aboveground plant organs (Figure 1). Hemp translocated more Cu and Ni in ABG than BGB than giant reed that instead showed the opposite for Cr (Table 3). TF showed the following crop ranking for the three HMs: Cr (giant reed 0.15 > hemp 0.05), Cr (hemp 0.46 > giant reed 0.07), and Cu (hemp 0.44 > giant reed 0.30). BCF values for ABG (Table 3) was in general similar among phytoremediation technique with giant reed showing higher BCF than hemp for Cr (0.062 vs. 0.003), Ni (0.04 vs. 0.12), and Cu (0.32 vs. 0.22). The only exception was observed in the BCF-ABG of Ni for hemp treated with EDTA (0.29) that was significantly higher than other treatments (0.04). EDTA increased significantly Cu concentration in belowground organs of giant reed while in hemp either aboveground and belowground organs had higher Cu concentration with EDTA addition than C and C+PGRP (Figure 1). EDTA addition increased significantly only Ni concentration in hemp leaves and giant reed rhizomes. Cr concentration in ABG is generally less affected by phytoremediation techniques. Only rhizomes of giant reed showed a significantly higher Cr concentration than C and C+EDTA. EDTA greatly enhances BCF of Ni and Cu in belowground organs of both crops especially (Table 3). HM element concentrations decreased differently in the plant organs of giant reed and hemp (Figure 1). Considering leaching in sediments cultivated with giant reed, levels of heavy metals in the leachate were differentially affected by EDTA (Supplementary Table 1). With EDTA, Ni and Cu leached easily after two applications showing, at the end of the experiment, significantly higher concentration of Ni (20.4 mg L^–1^) and Cu (17.9 mg L^–1^). Peak concentration of Cu in leachate was observed already 4 days after treatment while 16 DAT for Ni. Without EDTA, heavy metal concentration in leachates were very low on average (Cr: 2.68 ng L^–1^, Ni: 0.04 mg L^–1^, and Cu: 0.03 mg L^–1^). Bioaugmentation with PGPR never sustained HM leaching compared with sediment contaminated alone.

#### Heavy Metals Mass Balance and Uptake

Supplementary Figure 3 shows the HM mass balance for the hemp and giant reed experiments. At the time scale of the experiment, main Cu, Ni, and Cr remained in the sediment (99.7, 99.5, and 96.3%) and in the soil (>99%). A maximum of 1.45% of Cu, 0.72% of Ni, and 0.5% of Cr in the sediment was removed by giant reed treated with EDTA although at the same time 7.9% of Cu and 3% of Ni was lost with leaching. Hemp showed a lower HM removal from soil mass balance than giant reed with less variation among treatments (Supplementary Figure 3). An average of 30, 26, and 37‰ of Cu, Ni, and Cr, respectively, in soil was removed by hemp.

Overall ABG contributed very little to HM removal either in terms of mass balance (‰) (Supplementary Figure 3) and HM uptake at plant level (μg tissue^–1^) (Figure 2). The two plant micronutrients, Ni and Cu, showed very low whole plant uptake values (Ni: < 4 μg plant^–1^ and Cu: < 2 μg plant^–1^) for both crops grown in NC soil and sediment, respectively. In giant reed, compared with contaminated control (C), the significantly highest uptake values were observed in rhizomes with EDTA (Ni: 4.8 mg plant^–1^, Ni: 3.4 mg plant^–1^) and with PGPR either in leaves (Cr: 51 μg plant^–1^, Ni: 27 μg plant^–1^, Cu: 130 μg plant^–1^) and in stems (Ni: 41 μg plant^–1^, Cu: 137 μg plant^–1^). Roots of giant reed also contributed significantly to BGB HM mass balance (Figure 2 and Supplementary Figure 3). PGPR and EDTA significantly increased Cr and Cu root uptake in giant reed (Figure 2). In hemp, compared with the contaminated control (C), the significantly highest uptake values were observed for Cu in stems, leaves, and flowers with EDTA (950, 760, and 91 μg plant^–1^) and for Ni in stems with EDTA (2,800 μg plant^–1^).

### Plant Photosynthetic Performances

**FIGURE 3:**
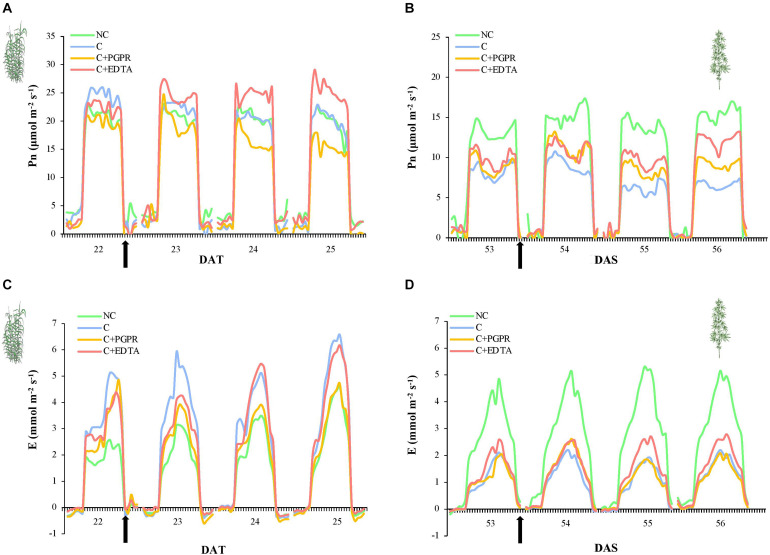
Whole-canopy net photosynthetic rate (Pn, μmol m^–2^ s^–1^) and transpiration rate (E, mmol m^–2^ s^–1^) of giant reed **(A)** and hemp **(B)** as affected by PGRP and EDTA application (black arrows). DAT, day after transplanting.

**FIGURE 4:**
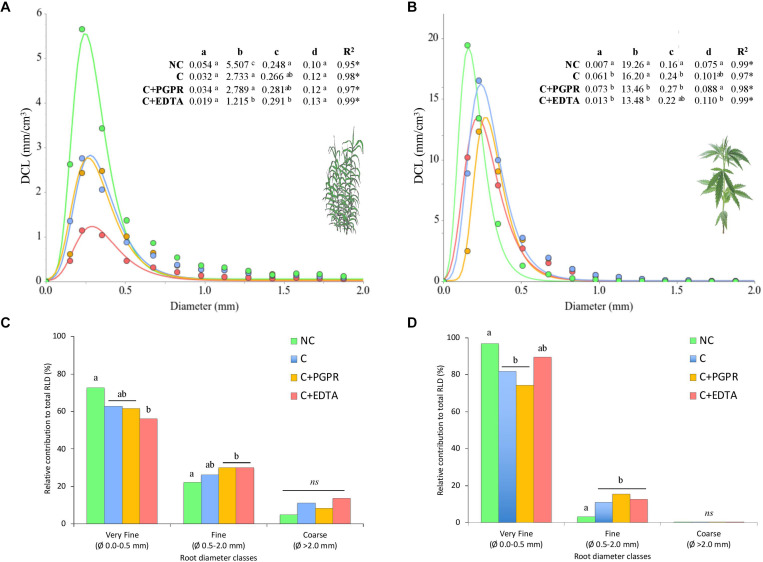
Diameter class length (DCL, mm cm^–3^) distribution of hemp **(A)** and giant reed **(B)** whole root systems and **(C**,**D)** relative contribution (%) of the very fine, fine, and coarse roots to the total root length density (RLD). Coefficients and statistics obtained from the regression of extreme value model are reported in the table where the statistical significance of the DCL curve parameters (*a*–*d*) were assessed through testing their standard errors using the t-statistics at *P* < 0.05. Different letters in graph **(C**,**D)** denote statistical differences (Tukey’s test, *P* = 0.05) among treatment for each root diameter classes.

The effects on photosynthetic performances (Pn and E) of HM pollution were more evident in hemp (Figures 3B,D) than in giant reed (Figures 3A,C). In fact, hemp plants grown in non-contaminated soil (NC) showed higher and constant Pn and *E*-values (13.9 and 3.5 mmol m^–2^ s^–1^, respectively) than hemp plants grown in contaminated soil (Figures 3B,D, *P* < 0.05). On the other hand, Pn of hemp plants grown in contaminated soil slightly decreased from an initial value of 8.3 until 6.6 μmol m^–2^ s^–1^ (*P* < 0.05). On 53 DAS, before PGPR and EDTA inoculation, no statistically significant differences were highlighted between the three treatments (Figures 3B,D). After 3 days from inoculation, differences in Pn become significant between PGPR and EDTA-inoculated plants (Figure 4B) with 9 and 12 μmol m^–2^ s^–1^, respectively. Transpiration rate was heavily affected by soil pollution: only hemp plants grown on non-contaminated soil reached on average *E*-values around 3.5 mmol m^–2^ i^–1^. No statistical differences resulted between treatments before and after the inoculation (Figure 3D).

In giant reed plants, soil pollution did not affect Pn neither before nor after the inoculation, remaining stable around 20.4 μmol m^–2^ s^–1^. Before the inoculation, no statistically significant differences in Pn were highlighted between PGPR and EDTA treatment. The 2nd day after inoculation, Pn of giant reed plants treated with PGPR significantly decreased Pn until 15.5 μmol m^–2^ s^–1^, whilse Pn of EDTA-treated plants increased until 25.5 μmol m^–2^ s^–1^ (Figure 3A, *P* < 0.05). Transpiration rate of plants grown on non-contaminated soil was not statistically different from E of PGPR-inoculated plants and lower than E of giant reed plant grown on contaminated soil. Pn of EDTA-inoculated plants slightly increased in response to the inoculation treatments from 3 to 4.4 mmol m^–2^ s^–1^ (Figure 3B, *P* < 0.05).

### Root-Microorganism Activity Interactions

Heavy metal contamination affected both in sediment and soil fine root biomass (FRB) and RLD of giant reed and hemp. Both crop yielded in non-contaminated matrices more FRB than in contaminated ones. EDTA significantly affected RLD of giant reed (*F* = 34, *P* = 0.001) showing a peak negative value on average of 0.5 cm cm^–3^. Significantly, RLD higher values were observed for C and PGPR (on average 1 cm cm^–3^) and NC (1.7 cm cm^–3^). A significant denser fine root system was observed in C treatments with hemp (average 3.66 cm cm^–3^) with no differences among them.

Diameter class length results indicate that the large majority (88.2% in giant reed and 99.2% in hemp) of the roots, expressed as RLD is composed of roots with a diameter lower than 2 mm (Figures 4C,D). Among these, very fine roots (0.0–0.5 mm) were more frequent than fine roots (0.5–2.0 mm), but the latter were the most affected by heavy metal contamination. In particular, EDTA application significantly decreases RLD of very fine roots of giant reed at the expense of fine roots. The extreme value model accurately described (average *R*^2^ of 0.98) the DCL distribution of the whole fine root system of giant reed and hemp (Figures 4A,B). Root system of both crops responded to heavy metal contamination by becoming thicker and shorter. Coefficients *a*, *b*, and *c* were significantly affected by NC and C treatments in both crops. Hemp roots thickened (*c* coefficient) from 0.16 mm with NC to an average value of 0.23 mm for contaminated soil treatments (Figure 4B). EDTA and PGPR in hemp significantly suppressed DCL at peack values (*b* coefficient) and increase the curve amplitude (width across the curve at half maximum—*d* parameter). For giant reed (Figure 4B), the model estimated that DCL peak was reached at a higher root diameter size in EDTA than in other treatment herbaceous crops (0.291 vs. 0.265 mm, respectively; *P* < 0.001) (Figure 4A). In particular, EDTA suppressed significantly DCL of these peak value (*b* parameter) to 1.2 cm cm^–3^ compared with 5.5 of NC and 2.7 and 2.8, respectively, of C and C+PGPR.

The results of soil EA (Supplementary Table 2) showed that HM contamination decreased significantly either of BS (-28% hemp and −37% giant reed) and RS (−39% hemp and −48% giant reed). In particular, RS of giant reed when cultivate on contaminated sediment showed a decrease compared with NC at 37, 38, 46, and 38%, respectively, for C-, N-, P-, and S-acquiring enzymes while esterases and microbial biomass were reduced at 34 and 12%, respectively. RS of hemp instead when cultivate on contaminated sediment showed a decrease compared with NC at 40, 32, 47, and 66%, respectively, for C-, N-, P-, and S-acquiring enzymes while esterases and microbial biomass were reduced of 55 and 32%, respectively. Highest reduction in EA and microbial biomass of RS were observed with EDTA where it reached values of −44 and −48% in hemp and −7 and −44% in giant reed. The highest reduction were observed for P- and S-acquiring enzymes in giant reed RS (−6 and −76%) and N- and P-acquiring in hemp (−55 and −56%).

**FIGURE 5:**
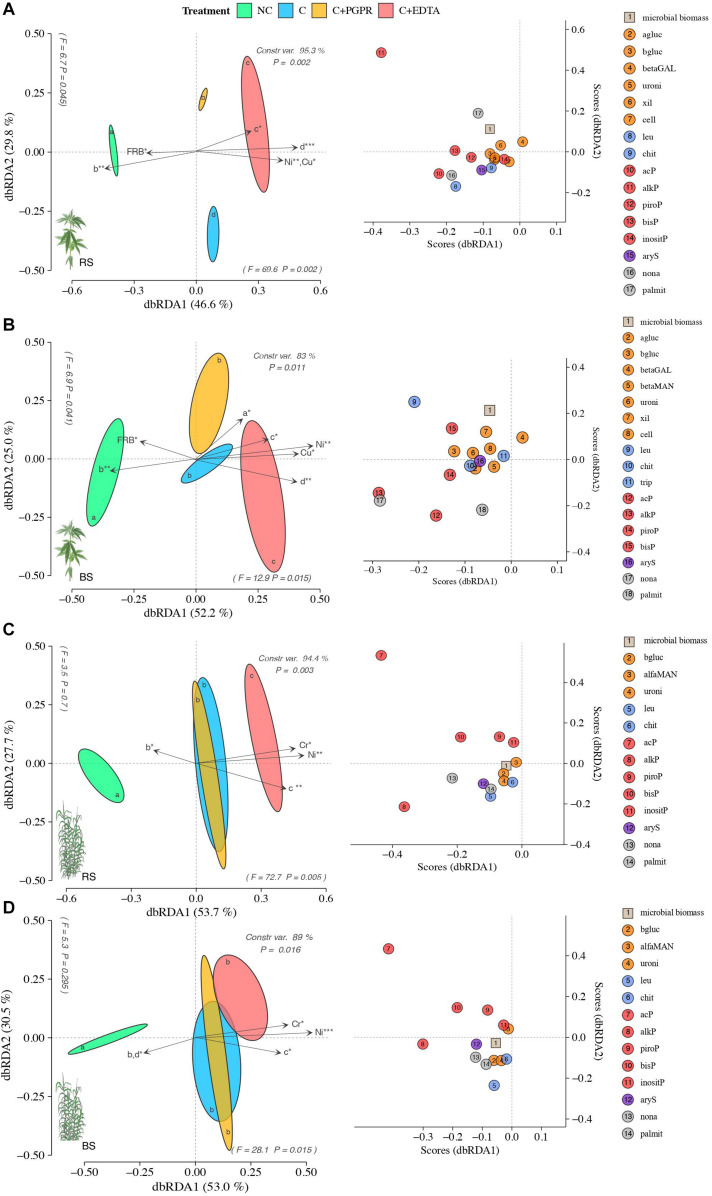
Distance-based redundancy analysis (dbRDA) plots showing shifts in enzyme activities of RS **(A**,**C)** and BS **(B**,**D)** of hemp **(A**,**B)** and giant reed **(C**,**D)** among treatments. Arrow indicates environmental variables with significance level (* < 0.05, ** < 0.001, *** < 0.001). Species scores corresponding to the dbRDA plots (coordinates for enzymes included in model) are reported in the scatter plots on the right. Letters within ellipses denote significant differences (Bonferroni’s test, *P* = 0.05) in EA similarity matrices among fertilizers as assessed by permutational multivariate analysis of variance (PERMANOVA).

Soil EA patterns in RS significantly differed among treatments more in hemp (*F* = 12, *P* = 0.002) than in giant reed (*F* = 8.9, *P* = 0.003) (Figures 5A,C). Similar but less pronounced separation along dbRDA axes were observed for BS of both crops. Relatively to RS, soil EA associated to hemp differed among NC and C treatments with separation along axes 1 (*F* = 69.6 and *P* = 0.002) accounting for 49.6% of the total variance and along axes 2 (*F* = 6.7 and *P* = 0.045) accounting for 29.8% of the total variance (Figure 5A). In hemp, soil EA patterns for C+EDTA were closer to each C and C+PGPR only in BS than those in BS (Figures 5A,B). PERMANOVA analysis (Supplementary Table 3) of EA and species score plot (Figures 5A,B) showed that EDTA significantly reduced C-, N-, and P-acquiring enzymes (alkP and leu) compared with contaminated control (C) while PGPR differed from C only for lower P- and S-acquiring enzymes (leu and bisP).

Similar effects of treatment on EA patterns differentiation were observed in giant reed, but different EA caused the horizontal differentiation in the dbRDA plots (Figures 5C,D). In the RS of giant reed EA differed among NC and C treatments with the separation along axes 1 (*F* = 72.7 and *P* = 0.052) accounting for 53.7% of the total variance (Figure 5C) while in BS C treatments did not differ from each other but only with NC with the separation along axes 1 (*F* = 28.1 and *P* = 0.015), accounting for 53% of the total variance (Figure 5D). PERMANOVA analysis (Supplementary Table 1) of EA and species score plot (Figures 5A,B) showed that EDTA in giant reed significantly reduced only N- and P-acquiring enzymes (acP, bisP, piroP, chit, leu) compared with the contaminated control (C) while PGPR did not differ from C for any EA group. dbRDA results from multivariate multiple regression on EA (arrows in Figure 5) indicate that the HM that had the highest influence on EA distribution in hemp were Ni and Cu (Figures 5A,B) and Ni and Cr in giant reed (Figures 5C,D). DCL curve parameters representative of ticker and longer root system (Figures 5C,D parameters) of hemp and giant reed were positively correlated with HM concentration and negatively correlated with EA. Denser root system represented by higher value of *b* parameter in NC treatment were instead positively correlated with EA.

### Bulk Soil and Rhizosphere Bacterial Diversity

A total of 9,021,165 raw reads were obtained for all soil/sediment analyzed, which were finally reduced to 8,086,439 after quality filtering. Average number of reads per sample was 89,849, and the average length was 300 bp in paired reads. Samples were rarefied to 10,000 sequences each, which was the abundance of the lowest populated sample: an average Good’s coverage of 86.5% (standard deviation 1) was found, indicating a good coverage of total bacterial diversity.

When samples were analyzed by means of hierarchical clustering of sequences taxonomically classified at the genus level, clear differences emerged for both giant reed (Supplementary Figure 4) and hemp experiments (Supplementary Figure 5). It is worth noting that not only the EDTA but also the inoculation with a single strain (PGPR theses) resulted in different bacterial communities already at time zero. Interestingly, the relative amount of sequences was also found to be classified as belonging to the *Enterobacter* genus (to which both inoculated strains belong) where higher in the PGPR treatments, thus indicating a significant enrichment due to the bacteria inoculation.

**FIGURE 6:**
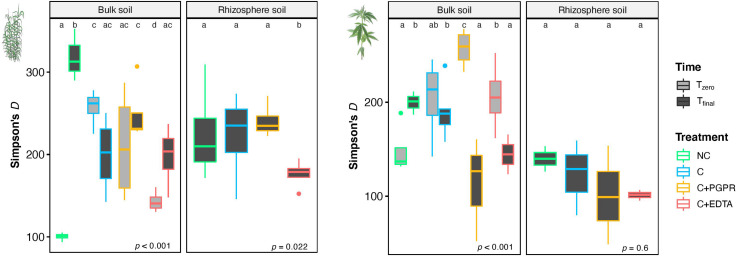
Mean values of Simpson’s index (*D*) in BS and RS of giant reed **(Left)** and hemp **(Right)** as affected by treatments and sampling time (*T*_*z*_, time zero sampling; *T*_*f*_, at the end of experiment). Different letters denote statistically different (Tukey’s test, *P* = 0.05) *D*-values among treatment in BS and RS for single crops.

Multivariate analyses on the total OTU matrixes show significant effects for sample type and for their interaction time × treatment terms (Figure 6). All tested effects were significant, with a percentage of variance ranging between 21.0% (hemp bulk soil) and 61.1% (giant reed BS). In agreement with the hierarchical clustering analyses, samples were forming separate groups, especially in the rhizosphere samples (Figure 6). In the case of giant reed, C+EDTA-treated samples were completely grouped apart, while C+PGPR were partly overlapping with C, while in hemp it was found that the C+PGPR samples formed a separate cluster from the other two C groups.

**FIGURE 7:**
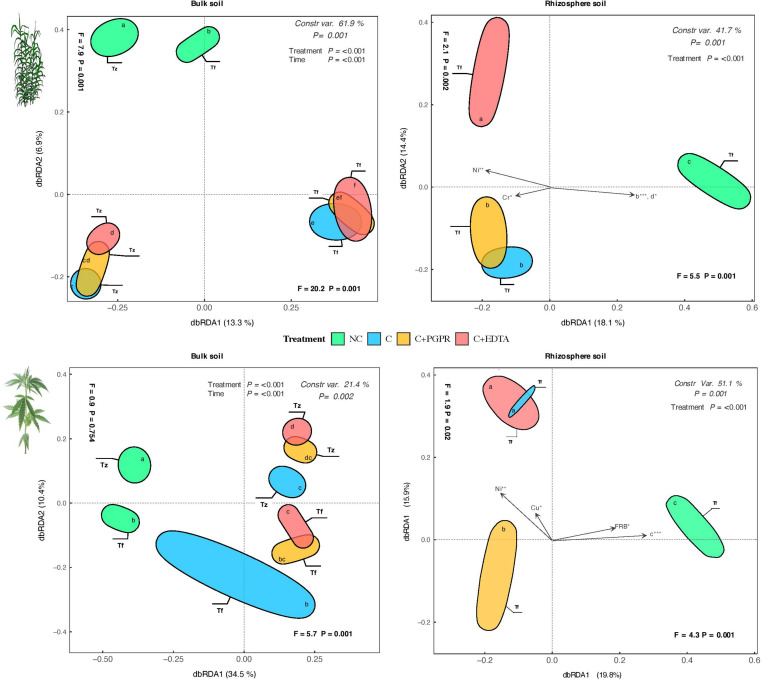
Distance-based redundancy analysis (dbRDA) plots showing shifts in microbial diversity (OUT) of RS and BS of hemp and giant reed among treatments and sampling time. Arrow indicates environmental variables with significance level (* < 0.05, ** < 0.001, *** < 0.001). Letters within ellipses denote significant differences (Bonferroni’s test, *P* = 0.05) in OUT’s similarity matrices among treatments/sampling time combination as assessed by permutational multivariate analysis of variance (PERMANOVA).

A number of significant differences were also found for α-diversity index, as depicted in Figure 7 for Simpson’s index. Focusing on rhizosphere samples, it was worth noting that in the case of giant reed, a significant reduction of diversity was found for C+EDTA as compared with C+PGPR, C, and NC. The same trend was observed for hemp, but with no statistical differences.

## Discussion

### Hemp and Giant Reed Phyto-Assisted Bioremediation Potential

In this study, two non-food crops (giant reed and hemp) were selected as candidate crops to reduce HMs of soil and sediments characterized by high concentration of Cr, Cu, and Ni. To do that, the phytoremediation potential of both traditional (crop alone) and assisted phytoremediation techniques (PGPR and chelating agent) was assessed. HM mass balance (Supplementary Figure 2), tolerance index (Table 1), and HM accumulation in plant organs (Figure 2) together indicated a good phytostabilization performance in giant reed and a moderate phytoextraction performance in hemp.

Heavy metal accumulation in hemp can be considered low for phytoextraction purposes under real field conditions (<100‰ in AGB, < 1% in BGB). Hemp showed a relatively low average Ni (25.1 mg kg^–1^) and Cu (8.4 mg kg^–1^) concentration and negligible Cr (<1 mg kg^–1^) concentration in AGB. Similar HM concentration values were found in other pot experiments with hemp grown on contaminated soil (Citterio et al., 2003; Angelova et al., 2004; Ahmad et al., 2016). The BCF values observed for Ni (0.3) and Cr (0.003) in AGB are indeed in line with those reported by Citterio et al. (2003). The low TF of HMs can be attributed to the low bioavailability of HMs in the soil (<2% as extracted with NH4NO_3_ 1:2.5 (w/v)—DIN 19730). Hemp yield was affected by the soil co-contaminants Ni (>500 mg kg^–1^) and Cu (>150 mg kg^–1^). Despite the use of EDTA slightly improved growth performance in terms of canopy photosynthesis, as observed also by Linger et al. (2005), a significant increase in HM uptake and translocation to aerial parts was observed. This confirms a good combination of hemp with EDTA (Citterio et al., 2003; Angelova et al., 2004; Ahmad et al., 2016) in particular for Cu uptake (3 times higher) but not for Ni (0.8 times higher). The mobilizing effects on soil HMs induced by EDTA (+10% in bioavailability of Ni and Cu) has to be considered, however, in view of its permanence in soil (Meers et al., 2005; Evangelou et al., 2007; Shahid et al., 2014) especially when high content of clay and soil organic matter are present, since they can both promote adsorption mechanisms of available HM.

Perennial energy crops have already been proposed by several authors as promising phytoremediation crop (Barbosa et al., 2015; Pandey et al., 2016). Our results on HM uptake and BCF indicate that giant reed in wet conditions accumulates most of the “extracted” HMs in belowground organs. The consistent BCF values observed in giant reed BGB (0.44, 0.56, and 0.84 for Cr, Ni, and Cu, respectively) confirm results by Barbosa et al. (2015) for Zn and Cr on soil and by Cristaldi et al. (2020) and Bonanno (2012) for Cr, Ni, and Cu in soil and sediment, respectively.

Our results on canopy photosynthesis (Figure 3) confirmed what already was found by several authors (Papazoglou et al., 2005, 2007; Fiorentino et al., 2017), as follows: giant reed tolerates well HM contamination because no statistically significant differences was observed between contaminated and not contaminated grown plants. Giant reed treated with PGPR showed contrasting results than EDTA which significantly increase both photosynthesis and transpiration. This results is unexpected for giant reed grown on contaminated sediment (Bonanno, 2012), but this can be partly explained by solubilization from sediments of entrapped plant nutrient.

The results on bioaugmented rhizoremediation of giant reed are promising for the following reasons: it tolerates from moderate to high level of a wide range of HMs (Fiorentino et al., 2017; Cristaldi et al., 2020), is a low-input perennial energy crop suitable for several marginal environment (Amaducci and Perego, 2015; Amaducci et al., 2016), and it performs equally in terms of phytoremediation as native species (Huguenot et al., 2015) such as common reed (*Phragmites australis*) if contaminated wastewaters are applied (Mirza et al., 2010; Kausar et al., 2012). From the comparison of bioaugmentation with PGPR and addition of EDTA, it emerged clearly that, considering field application and from an environmental point of view, microbial inoculum seems preferable over chelating agents. Although EDTA application showed an increase in the uptake of Ni and Cu (two known HMs for their mobility), but not in TF, the enrichment of leachates with HMs raise concerns over EDTA application in open environment conditions, especially for potential contamination of groundwaters, as already pointed out by other authors (Evangelou et al., 2007; Yang et al., 2012; Shahid et al., 2014). In particular, other chelating agents have been proposed to treat contaminated matrices with giant reed (Yang et al., 2012) and hemp (Meers et al., 2005). Alternatively, addition of chelating agents can be successfully performed with perennial plant in small stormwater basins connected to discharge areas of industrial sites (Huguenot et al., 2015).

Bioaugmentation with PGPR showed interesting results when combined with giant reed. PGPR increased the accumulation of Cr and Cu in rhizome of giant reed and enhanced the TF of these metals in AGB. Although PGPR decreased net photosynthesis in giant reed, we observed an increase of Cr, Ni, and Cu uptakes in leaves and Ni and Cu in stems. Our results showed that the PGPR strains selected (*Enterobacter* spp.) are not inhibited by HM contamination under *in vitro* conditions. There are several evidences that *Enterobacter* spp., and in particular *Enterobacter asburiae* shows tolerance genes to HMs (Nguyen et al., 2019). A strong resistance to heavy metals was reported for *Enterobacter* spp. found in contaminated soil and sediments (Neeta et al., 2016; Chen et al., 2017; Sharma et al., 2020). Moreover, this Gram-negative enteric bacteria have already been successfully inoculated to alleviated HM stress in other crops: soybean (Kang et al., 2015), rice (Mitra et al., 2018), and hyperaccumulator plants (Whiting et al., 2001). In giant reed, the use of other PGPR are documented for their HM biosorption capacity such as *Agrobacterium* spp. (Guarino et al., 2020) or *Bacillus* spp. (Sarathambal et al., 2017). The use of microorganism to alleviate HM stress of hemp is more focused toward AMF (Citterio et al., 2005) while PGPR associated to hemp plant growth improvement are more common (Pagnani et al., 2018; Lyu et al., 2019). Interestingly, the two PGPR strains used in this study were selected for both high HM biosorption and P solubilization abilities.

### Insights From Plant-Soil-Microbe Interactions in Microbial-Assisted Phytoremediation

In this work, we investigated the effects of three microbial-assisted phytoremediation strategies of HM-contaminated soils and sediments on fine root system morphology and bacterial community structure and activity.

Although giant reed may appear suitable for phytostabilization, based on its HM tolerance, exposure to HMs drastically impairs its root distribution (Figure 4). This is even more evident in hemp grown on contaminated soil. A general reduction of RLD associated to HMs is a known fact for many crops (Keller et al., 2003; Peer et al., 2006; Ostonen et al., 2007). Our result provided for the first-time evidence of the effect of HM contamination of soil and sediment on root diameter class length distribution of hemp and giant reed. HM contamination resulted in ticker and shorter root system, as shown by data on relative contribution to total RLD and root DCL curve distribution. Fine root system morphology of these crops have been characterized under field condition in non-contaminated soil (Amaducci et al., 2008; Chimento and Amaducci, 2015).

Interestingly, RLD of very fine roots of giant reed were stimulated more by PGPR than EDTA. Stimulation of root and shoot length by microbial inoculations was also observed under HMs stress by other authors (Liu L. et al., 2018; Pagnani et al., 2018), and is in line with the role of *Enterobacter* spp. in improving root systems through the production of phytohormones (Naveed et al., 2014). This outcome is also in agreement with the contemporary enhancement of growth and HM uptake of giant reed under Ni and Cu contaminations in sediments. The increase in RLD of very fine roots can be attributed to the plant growth-promoting traits possessed by the inoculated microorganisms.

The HM-induced alterations of fine root system morphology are often reported to be metal and species specific (Lambrechts et al., 2014). Despite that the environmental matrices used in this study are contaminated by different HMs, a larger cumulative root density/aboveground biomass ratio as suggested by Keller et al. (2003), together with similar relative proportion of fine roots to contaminated control (C), are two root traits associated with PGPR addition that helped increase HM uptake by giant reed. We suggest therefore that DCL curve distribution can be used successfully as an indicator of HM phytoextraction ability of perennial crops, but this hypothesis has to be further tested under real field conditions. Another relevant finding of our study is that DCL curve parameters representative of the thickest and shortest root system (*c* and *d* parameters) of hemp and giant reed were negatively correlated with soil EA and positively correlated with HM levels (Figure 5). The negative effects of single- or multi-HM pollution on soil EA is well known (Burges et al., 2015; Xian et al., 2015). A recent meta-analysis (Aponte et al., 2020) on HM effect on soil EA showed that HM contamination linearly reduce the activities of extracellular enzymes involved in S (−60%) and N (−30%) cycling two–three times more than those involved in P and C cycling (−10%). Our findings showed a general higher reduction of EA under HM contamination in RS than BS. In rhizosphere soil, C-, N-, P-, and S-acquiring enzymes were on average reduced by 38, 35, 47, and 52%, respectively. In particular, along root phosphomoestares (alkP), phosphomoestares (piroP), and arylsulfatase (aryS) were the EA most impacted by HM contamination. This confirms what was observed spatially with zymography under HM stress by Ma et al. (2018). Fine roots are on hot spot for microbial activity (Spohn et al., 2013; Kuzyakov and Blagodatskaya, 2015) and more diverse and species-rich microbial community (Pervaiz et al., 2020). Fine roots play also an important role in managing the accessibility of metal ions to plant roots (Srivastava et al., 2017). Multivariate analyses (dbRDA) of EA patterns clearly indicated how bioaugmentation with PGPR and addition of EDTA shaped differentially microbial community either in terms of activity (Figure 5) and diversity (Figure 6).

A consistent indirect effect of HM levels on the soil microbiome (diversity and activity) mediated by plant response in terms of root growth (DCL distribution) was observed for both crops. HMs affected first root architecture, with fine roots thicker and longer, and a result of these changes occurred at root level the microorganism colonizing root systems have been consequentially affected (EA and OTU diversity). Considering the results obtained by Illumina OTU analyses, it is worth noting that the dbRDA pattern of OTU distribution (Figure 6) was quite consistent with the ones obtained on soil EA (Figure 5), thus showing a good agreement between the response of microbial communities both in terms of structure and activity measurements. It is also worth noting that EDTA had a more detrimental effect on bacterial diversity as compared with PGPR: this outcome points once again to a higher acceptability and environmental sustainability of the bioaugmentation approach as compared with the addition of chelating agent.

Despite that results on HM removal efficiency are promising especially for giant reed, the detrimental effect of HMs on root system morphology is the main cause for the lower activity and diversity (Figure 7) of microbial communities in RS and BS. Root DCL distribution, represented by the coefficient of extreme value model proposed by Zobel et al. (2007), might be suggested as a key ecological trait to understand crop-specific effect of HMs on microbial activity and diversity.

To conclude, insights from plant-soil-microbe interactions under HM contamination were addressed for two important non-food high-yielding crops. Such knowledge might help to improve phytoremediation on target site, e.g., by shortening the time needed to reach the HM threshold for public use. In order to guide selection of even more efficient phyto-assisted bioremediation technologies for other contaminated sites, future research should target (1) non-food crops with good phytoaccumulation potentials and (2) improved understanding of a wide range of plant mechanism affected by PGPR and *vice versa*, in order to improve the effectiveness of crop-microbe interactions in reducing HM levels.

## Data Availability Statement

The datasets presented in this study can be found in online repositories. The names of the repository/repositories and accession number(s) can be found below: https://www.ncbi.nlm.nih.gov/, BioProject PRJNA687540.

## Author Contributions

AFe, AFr, SA, and EP: conceived and designed the experiment. AFr and AFe: managed the pot experiment. AFe and EP: performed soil sampling. GS: performed PGPR selection. GS and ET: performed microbiological analysis. AFr: performed plant growth analyses and performed root measurements. FF: performed soil enzymatic activities analysis. GB and MF: performed HM analysis. AFe: analyzed the data. SA and EP: contributed reagents/materials. AFe, SA, and EP: wrote the manuscript. All authors contributed to the article and approved the submitted version.

## Conflict of Interest

FF was employed by company SOLIOMICS srl. The remaining authors declare that the research was conducted in the absence of any commercial or financial relationships that could be construed as a potential conflict of interest.
