# Supplementary material for: Bioaugmented Phytoremediation of Metal-Contaminated Soils and Sediments by Hemp and Giant Reed
Source: Front Microbiol. 2021 Apr 20;12:645893. doi: 10.3389/fmicb.2021.645893 (PMC8096354; doi:10.3389/fmicb.2021.645893)
Supplement: Supplementary file 1 [file Table_1.docx]

Supplementary Material

**Bioaugmented bioremediation of metal-contaminated soils and sediments by hemp and giant reed**

Andrea Ferrarini^1*^, Alessandra Fracasso^1^, Giulia Spini^2^, Flavio Fornasier^3,4^, Eren Taskin^2^, Maria Chiara Fontanella^2^, Gian Maria Beone^2^, Stefano Amaducci^1^, Edoardo Puglisi^2^

^1^ Department of Sustainable Crop Production, Università Cattolica del Sacro Cuore, Via Emilia Parmense 84, 29122, Piacenza, Italy

^2^ Department for Sustainable Food Process, Università Cattolica del Sacro Cuore, Via Emilia Parmense 84, 29122, Piacenza, Italy

^3^ CREA – Centro Viticoltura ed Enologia, Via Trieste 23, Gorizia, 34170 , Italy

^4^ SOLIOMICS srl, Via del Cotonificio, 129/B 33100 Udine, Italy

**Figure S1** Experimental design of the greenhouse pot experiment under controlled conditions

**Table S1** Effect of EDTA application on leachate Cr, Ni and Cu concentrations in the giant reed leaching experiment; brackets: ±S.E. DAS: days after sowing; DAT: days after EDTA treatments. DAT: days after transplanting. DAA_n_: days after *n-th* EDTA application. Different lowercase letters indicate significant differences (Tukey’s test P<0.05) among treatments for each element over time. ANOVA has been run separately for giant reed and hemp.

| **DAT** | **DAA_1_** (DAT:22) | **DAA_2_** (DAT:53) | **Cr** (ng L^-1^) | | | **Ni** (mg L^-1^) | | | **Cu** (mg L^-1^) | | |
| --- | --- | --- | --- | --- | --- | --- | --- | --- | --- | --- | --- |
|  |  |  | **C** | **PGPR** | **EDTA** | **C** | **PGPR** | **EDTA** | **C** | **PGPR** | **EDTA** |
| 17 | - | - | 2.16 a | 2.56 a | 2.03 a | 0.03 a | 0.03 a | 0.03 a | 0.02 a | 0.03 a | 0.02 a |
|  |  |  | (0.18) | (0.11) | (0.25) | (0.00) | (0.01) | (0.00) | (0.00) | (0.01) | (0.00) |
| 26 | 4 | - | 2.29 a | 2.10 a | 2.66 a | 0.03 a | 0.02 a | 6.85 a | 0.01 a | 0.01 a | 8.18 b |
|  |  |  | (0.37) | (0.27) | (0.31) | (0.01) | (0.00) | (1.55) | (0.00) | (0.00) | (1.82) |
| 38 | 16 | - | 3.14 a | 3.85 a | 2.92 a | 0.03 a | 0.03 a | 8.64 b | 0.02 a | 0.05 a | 8.78 b |
|  |  |  | (0.60) | (0.20) | (0.37) | (0.01) | (0.01) | (1.49) | (0.01) | (0.01) | (2.18) |
| 60 | 38 | 7 | 2.63 a | 2.68 a | 3.06 a | 0.05 a | 0.12 a | 20.42 c | 0.01 a | 0.06 a | 17.84 c |
|  |  |  | (0.56) | (0.62) | (0.23) | (0.02) | (0.02) | (2.89) | (0.00) | (0.02) | (2.55) |

**Table S2** Average values of microbial biomass (ng dsDNA g soil) and soil enzymatic activities (nmol MUF or AMC h^-1^ g_soil_ ^-1^) in bulk (BS) and rhizosphere (RS) soil of giant reed and hemp at the end of experiment as affected by treatments. Enzymatic activities abbreviations: agluc: α-glucosidase, bgluc : β-glucosidase, alfaGAL: α-galactosidase, betaGAL: β-galactosidase, alfaMAN: α-mannosidase, betaMAN: β-mannosidase, uroni: β-D-glucuronidase, cell: β-1,4-glucanase, xilo: β-1,4-xylanase chit: N-acetyl-b-D-glucosaminidase, leu: leucine amino-peptidase, tryp: trypsin-like protease, acP and alkP: acid and alkaline phosphomonoesterase, bisP: phosphodiesterase, piroP: pyrophosphodiesterase, inositP: inositol-P phosphatase, aryS: arylsulfatase, nona: nonanoate esterase, palmit: palmitate esterase.

| **Crop** | **Soil** | **Treatment** | **Microbial biomass** | **C- acquiring enzymes** | | | | | | | | |
| --- | --- | --- | --- | --- | --- | --- | --- | --- | --- | --- | --- | --- |
|  |  |  |  | **agluc** | **bgluc** | **alfaGAL** | **betaGAL** | **alfaMAN** | **betaMAN** | **uroni** | **xil** | **cell** |
| Giant Reed | BS | NC | 2.16 | 0.16 | 3.05 | 0.34 | 0.57 | 0.42 | 0.90 | 2.66 | 0.64 | 0.26 |
|  |  | C | 1.87 | 0.13 | 2.11 | 0.27 | 0.42 | 0.28 | 0.43 | 2.30 | 0.46 | 0.17 |
|  |  | C+EDTA | 1.09 | 0.13 | 1.93 | 0.27 | 0.42 | 0.29 | 0.54 | 1.94 | 0.42 | 0.16 |
|  |  | C+PGPR | 1.52 | 0.15 | 2.30 | 0.29 | 0.46 | 0.32 | 0.49 | 2.43 | 0.49 | 0.18 |
|  | RS | NC | 4.05 | 0.30 | 4.64 | 0.59 | 0.94 | 0.63 | 1.64 | 4.64 | 0.89 | 0.31 |
|  |  | C | 3.38 | 0.18 | 2.54 | 0.36 | 0.56 | 0.37 | 0.96 | 2.82 | 0.54 | 0.19 |
|  |  | C+EDTA | 2.18 | 0.16 | 2.56 | 0.33 | 0.52 | 0.35 | 0.92 | 2.56 | 0.49 | 0.17 |
|  |  | C+PGPR | 2.75 | 0.20 | 2.81 | 0.39 | 0.61 | 0.41 | 1.07 | 3.03 | 0.59 | 0.20 |
| Hemp | BS | NC | 10.91 | 0.74 | 8.60 | 0.35 | 0.55 | 0.42 | 0.90 | 3.75 | 1.21 | 0.53 |
|  |  | C | 9.80 | 0.32 | 6.86 | 0.27 | 0.63 | 0.27 | 0.45 | 3.03 | 0.68 | 0.43 |
|  |  | C+EDTA | 8.82 | 0.13 | 4.38 | 0.26 | 0.99 | 0.28 | 0.55 | 1.92 | 0.62 | 0.16 |
|  |  | C+PGPR | 12.27 | 0.15 | 5.96 | 0.29 | 0.84 | 0.30 | 0.47 | 3.02 | 0.93 | 0.43 |
|  | RS | NC | 22.22 | 1.30 | 11.64 | 0.59 | 0.94 | 0.63 | 1.64 | 6.17 | 1.67 | 0.64 |
|  |  | C | 15.87 | 0.43 | 9.04 | 0.36 | 0.83 | 0.37 | 0.96 | 4.32 | 0.63 | 0.48 |
|  |  | C+EDTA | 14.65 | 0.16 | 6.06 | 0.33 | 1.20 | 0.35 | 0.92 | 2.97 | 0.59 | 0.17 |
|  |  | C+PGPR | 19.50 | 0.20 | 8.56 | 0.39 | 1.11 | 0.41 | 1.07 | 3.78 | 0.64 | 0.49 |

| **Crop** | **Soil** | **Treatment** | **N- acquiring enzymes** | | | **P- acquiring enzymes** | | | | | **S- acq** | **Esterases** | |
| --- | --- | --- | --- | --- | --- | --- | --- | --- | --- | --- | --- | --- | --- |
|  |  |  | **leu** | **chit** | **trip** | **acP** | **alkP** | **piroP** | **bisP** | **inositP** | **aryS** | **nona** | **palmit** |
| Giant Reed | BS | NC | 6.60 | 0.98 | 0.57 | 51.69 | 72.68 | 1.64 | 9.17 | 0.29 | 2.67 | 23.07 | 1.70 |
|  |  | C | 6.37 | 0.95 | 0.62 | 27.19 | 45.56 | 0.97 | 4.50 | 0.20 | 1.03 | 19.65 | 0.90 |
|  |  | C+EDTA | 4.26 | 0.62 | 0.54 | 26.75 | 48.23 | 0.99 | 4.47 | 0.20 | 0.87 | 17.49 | 0.60 |
|  |  | C+PGPR | 5.26 | 0.77 | 0.56 | 27.16 | 53.12 | 0.71 | 4.70 | 0.14 | 0.96 | 17.98 | 0.78 |
|  | RS | NC | 10.58 | 1.39 | 0.81 | 116.86 | 163.43 | 3.60 | 20.55 | 0.65 | 5.89 | 53.23 | 3.76 |
|  |  | C | 7.61 | 1.12 | 0.73 | 59.46 | 102.45 | 2.17 | 9.59 | 0.47 | 2.39 | 31.58 | 1.50 |
|  |  | C+EDTA | 4.54 | 0.67 | 0.58 | 44.43 | 81.38 | 1.62 | 7.33 | 0.35 | 1.44 | 27.20 | 0.91 |
|  |  | C+PGPR | 6.80 | 0.90 | 0.65 | 60.40 | 113.67 | 1.93 | 10.58 | 0.34 | 2.14 | 30.39 | 1.37 |
| Hemp | BS | NC | 18.06 | 4.65 | 0.96 | 39.42 | 127.64 | 4.29 | 11.84 | 0.69 | 8.29 | 65.05 | 10.00 |
|  |  | C | 14.64 | 3.93 | 1.04 | 34.92 | 93.69 | 2.41 | 6.48 | 0.60 | 5.50 | 42.28 | 8.19 |
|  |  | C+EDTA | 7.43 | 2.48 | 0.77 | 27.98 | 90.95 | 1.58 | 7.52 | 0.33 | 5.99 | 40.39 | 8.74 |
|  |  | C+PGPR | 14.83 | 3.11 | 0.97 | 28.86 | 97.94 | 1.66 | 8.36 | 0.63 | 5.95 | 43.21 | 8.33 |
|  | RS | NC | 27.08 | 6.68 | 1.51 | 89.36 | 260.93 | 9.35 | 30.55 | 1.57 | 18.79 | 97.48 | 23.62 |
|  |  | C | 17.61 | 4.62 | 0.89 | 59.46 | 147.45 | 3.68 | 15.42 | 1.02 | 12.39 | 72.21 | 13.14 |
|  |  | C+EDTA | 9.67 | 2.67 | 0.66 | 49.43 | 151.61 | 2.62 | 13.12 | 0.54 | 10.25 | 59.70 | 13.83 |
|  |  | C+PGPR | 16.12 | 3.65 | 0.84 | 60.40 | 186.17 | 4.06 | 17.43 | 0.98 | 10.36 | 72.49 | 15.42 |

**Table S3** Summary of significant differences (Bonferroni’s test, P: 0.05) in enzyme activities (grouped by element) among treatments for giant reed and hemp as assessed by Permutational multivariate analysis of variance (PERMANOVA).

|  | **Treatment** | **Rhizosphere soil** | | | | |  | | | **Bulk soil** | | | | |
| --- | --- | --- | --- | --- | --- | --- | --- | --- | --- | --- | --- | --- | --- | --- |
|  |  | **C** | **N** | **P** | **S** | **ester** | |  | **C** | | **N** | **P** | **S** | **ester** |
| 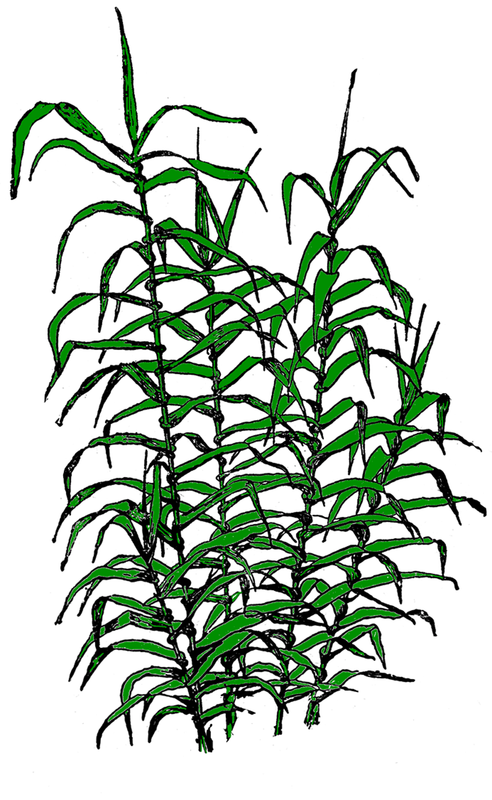 | NC | a | a | a | a | a | |  | a | | a | a | a | a |
|  | C | b | b | b | b | b | |  | b | | b | b | b | b |
|  | C+PGPR | b | bc | b | b | b | |  | ab | | ab | b | b | b |
|  | C+EDTA | b | c | c | b | b | |  | b | | b | b | b | b |
| 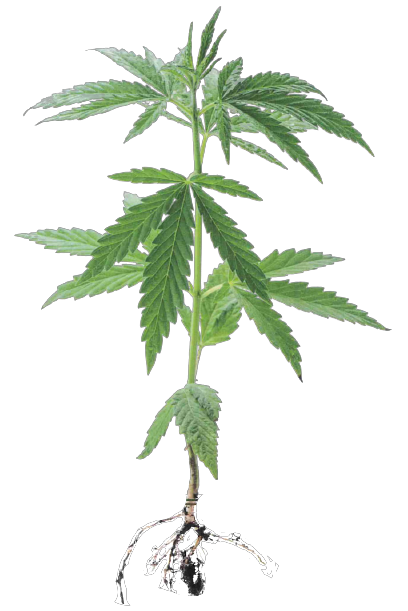 | NC | ab | a | a | a | a | |  | a | | a | a | a | a |
|  | C | b | b | b | b | b | |  | b | | ab | b | b | b |
|  | C+PGPR | b | b | c | c | ab | |  | b | | b | b | b | bc |
|  | C+EDTA | c | c | d | bc | b | |  | b | | c | b | b | c |


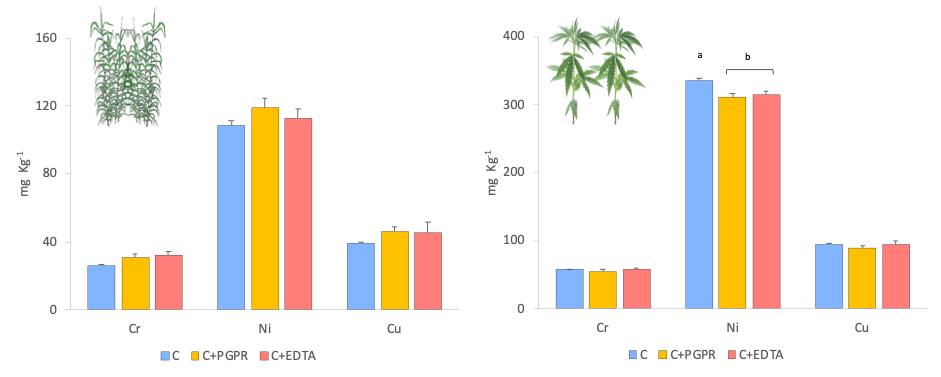


**Fig. S2** Average of soil total HM concentration at the end of experiment .

**Fig S3** HMs mass balance (% and ‰) for giant reed and hemp at the end of experiment

**Figure S4** Hierarchical clustering at the genus level of sequences from giant reed experiment performed on contaminated sediment. Clusters were identified with the average linkage algorithm for taxa that contributed at least 5% to a single sample. BS: bulk sediment, RS: rhizosphere sediement, NC: sediment not contaminated, C: contaminated sediment, C + PGRP: contaminated sediment treated with PGPR, C + EDTA: contaminated sediment treated with EDTA.

**Figure S5** Hierarchical clustering at the genus level of sequences from hemo experiment performed on contaminated soil. Clusters were identified with the average linkage algorithm for taxa that contributed at least 5% to a single sample. BS: bulk soil, RS: rhizosphere soil, NC: soil not contaminated, C: contaminated soil, C + PGRP: contaminated soil treated with PGPR, C + EDTA: contaminated soil treated with EDTA.
